# Supervisor Future Orientation and Humble Leadership: The Role of Supervisor Outcome Dependence and Prevention Focus

**DOI:** 10.3390/bs16040508

**Published:** 2026-03-28

**Authors:** Qin Xu, Miaomiao Sun, Hongjiang Lv, Shuming Zhao, Shichang Dong

**Affiliations:** 1 School of Economics & Management, Southeast University, Nanjing 211189, China; qin1985@hotmail.com (Q.X.);; 2 School of Business, Nanjing University, Nanjing 210093, China

**Keywords:** humble leadership, future orientation, outcome dependence, prevention regulatory focus

## Abstract

While extensive research has investigated the consequences of humble leadership, its formation remains understudied. Integrating power dependence theory and regulatory focus theory, we propose a model in which supervisor outcome dependence serves as a mediator and supervisor prevention focus works as a moderator in the relationship between supervisor future orientation and subordinates’ perceptions of humble leadership. Our results from a multiwave, multisource field study indicate that supervisor future orientation positively influences humble leadership perceptions through supervisor outcome dependence. Moreover, this indirect effect is stronger among supervisors with high prevention focus. This study concludes by discussing the theoretical and practical implications for leadership development.

## 1. Introduction

Humility has long been esteemed as a fundamental virtue, deeply embedded in diverse philosophical and religious traditions (Jia et al., 2025; Owens et al., 2013). In recent decades, however, a proliferation of moral scandals and ethically questionable corporate decisions has reinforced the critical need for humility in leadership (Zhong et al., 2023). Humble leadership is defined by the ability to assess oneself accurately, an appreciation of others’ strengths and contributions, and a willingness to embrace new ideas and feedback (Owens et al., 2013). Research has shown that humble leadership can lead to a wide range of favorable outcomes for followers, including heightened job satisfaction (Chandler et al., 2023), enhanced voice (Zhou & Chen, 2024), and greater creativity (Ye et al., 2020). Given these benefits, cultivating humble leadership in supervisors has become imperative for both researchers and practitioners (Wen et al., 2025).

Previous research on the formation of humble leadership has found that a leader’s characteristics (e.g., growth mindset), follower factors (e.g., competence), and contextual factors (e.g., significant-other activation) can all influence its development(Kelemen et al., 2023; Wang et al., 2018, 2024). Considering the significant role of leaders’ personalities in shaping their leadership styles (W.-H. Zhang et al., 2014), we propose that leaders with different future orientations, defined as a behavioral tendency to focus on future or distant consequences rather than immediate ones (Strathman et al., 1994), may exhibit varying levels of humble leadership behaviors, because of their differing attitudes towards future consequences during collaborative task execution. However, the specific process through which a leader’s temporal perspective translates into humble behavior remains a black box. Furthermore, a recent review by Kelemen et al. (2023) has called for research on what leader personal factors predict humble leadership (Wen et al., 2025). To address this gap and move beyond merely identifying new antecedents, we draw on power dependence theory to develop and test a novel explanatory mechanism linking a supervisor’s future orientation on subordinates’ perceptions of humble leadership.

Power dependence theory suggests that in dyadic relationships, one party’s power over another is inversely related to its dependence on the other. Dependence arises when an actor’s ability to achieve desired outcomes relies on critical resources controlled by another. As a result, the dependent actor is motivated to reduce this dependence and regain power (Emerson, 1962). In the workplace, supervisors with a strong future orientation may actively seek support and resource investment from their subordinates to secure long-term goals, and tend to increase their outcome dependence on subordinates. To reduce such dependence and strengthen their future power, these supervisors are likely to adopt a humble leadership style-acknowledging their limitations, valuing others’ strengths, and encouraging subordinates to contribute meaningfully. Therefore, we propose that supervisors’ outcome dependence on subordinates mediates the relationship between their future orientation and their display of humble leadership.

As noted by Kelemen et al. (2023), it is crucial to investigate the contingency factors that impact the emergence of a supervisor’s humble leadership. Thus, we further explore when a supervisor’s future orientation exerts an influence on subordinates’ perceptions of humble leadership through supervisor outcome dependence. According to regulatory focus theory, individuals with a strong prevention focus are more likely to avoid negative outcomes and failure in goal achievement (Higgins, 1998). Combining regulatory focus theory with power dependence theory, we propose that highly future-oriented supervisors are more likely to rely on the investments and contributions of subordinates to mitigate risks and achieve their future goals, making them more willing to lower their stature and behave humbly (Carnevale et al., 2019). Accordingly, we expect that the relationship between supervisor future orientation and humble leadership through supervisor outcome dependence will be stronger when supervisor prevention focus is relatively high.

**Figure 1:**
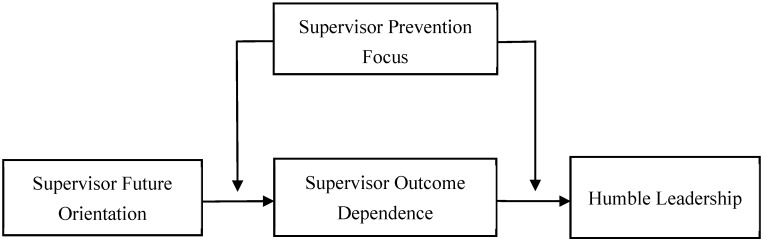
The Conceptual Model.

Taken together, drawing on power dependence and regulatory focus theories, we develop a theoretical model as presented in Figure 1. This paper is structured as follows. First, we review the theoretical background and develop hypotheses. Next, we describe the method, sample, and analytical procedures of our multi-wave, multi-source field study. We then present the results and, in the discussion section, elaborate on their theoretical and practical implications, address limitations, and suggest future research directions. This study makes several contributions to the existing literature. First, our exploration of the effect of supervisor future orientation on humble leadership enriches the antecedents of humble leadership in terms of personality traits (Wang et al., 2018; J. Yang et al., 2019). Second, it provides a new perspective to explain how a supervisor’s humble leadership is formed. Our study is based on power dependence theory and emphasizes the crucial role of a leader’s outcome dependence on subordinates in the relationship between supervisor future orientation and humble leadership. Finally, prior studies on the moderators of humble leadership development have focused on leader relational identity, authority centralization (Peng et al., 2020; Wang et al., 2024). This study expands the understanding of boundary conditions by examining the moderating role of supervisor prevention focus.

## 2. Theoretical Background and Hypothesis Development

### 2.1. Power Dependence Theory

Humility has been recognized as an important virtue for centuries. A humble individual is not only free from narcissism and arrogance, but also possesses an accurate self-assessment, a willingness to embrace new ideas and feedback, and a sense of transcendence (Owens et al., 2013). Over the past decade, organizational scholars have discovered that leader humility leads to a wide range of positive consequences, including team learning, employee job satisfaction, and creativity (Bahadur & Ali, 2023; Chandler et al., 2023; Jia et al., 2025). However, research on the antecedents of leader humility is considerably smaller in volume compared to studies focusing on its outcomes. A recent review by Kelemen et al. (2023) has called for further investigation into leader individual factors that predict humble leadership and the moderators of these antecedents. In response to this call, we argue that a leader’s future orientation is a key antecedent of humble leadership, as this trait is particularly critical in today’s rapidly changing environment (Guo et al., 2022), enabling leaders to engage in effective self-regulation (Mohammed & Marhefka, 2020) and better prepare for future outcomes (W.-H. Zhang et al., 2014).

Power dependence theory posits that an actor is motivated to engage in exchanges with another party in order to obtain resources and achieve the goal (Emerson, 1962). In the context to leadership, a supervisor and a subordinate form such an exchange pair (G. Zhang et al., 2020). A supervisor depends on subordinates because they control critical resources essential for group success, which ultimately fall under the supervisor’s responsibility. This dependency is inversely related to the leader’s power over subordinates. Power refers to the leader’s ability to secure favorable outcomes (e.g., promotion, rewards, compensation) in an exchange relationship (Emerson, 1962). A leader strives to increase their power while reducing their dependence. Therefore, power dependence theory offers a suitable framework for explaining how a supervisor’s future orientation leads to humble leadership. Highly future-oriented supervisors tend to prioritize achieving long-term outcomes and are motivated to accumulate the resources necessary to achieve these goals, which aligns closely with the logic of power dependence theory.

### 2.2. Supervisor Future Orientation and Outcome Dependence

Future orientation refers to an individual’s relatively stable belief in the value of investing in future resources (Strathman et al., 1994). It is considered a key determinant of self-regulation, goal setting, and achievement (Mohammed & Marhefka, 2020). Individuals with a strong future orientation are more motivated to build resources for an ideal future and take proactive steps toward achieving their desired outcomes. The majority of studies have found that employee future orientation is linked to increased performance (Lee et al., 2017), enhanced work proactivity (Lichtenthaler & Fischbach, 2019) and higher career adaptability (Y. Yang et al., 2024). A few researchers have shifted their focus and found that leaders with a high future orientation were more likely to clarify the future (Guo et al., 2022), foster growth and stimulate learning among their followers (W.-H. Zhang et al., 2014). Given the positive impact of individual future orientation on organizational adaptation in today’s VUCA (i.e., volatility, uncertainty, complexity, ambiguity) environment (Y. Yang et al., 2024), it is necessary to explore what kind of consequences future-oriented leaders will lead their groups to.

A supervisor’s outcome dependence refers to the extent to which a supervisor’s work outcomes are determined by subordinates’ contributions (Johnson & Johnson, 1989). This dependence can be heightened by leaders’ future orientation for two reasons. First, from power dependence theory (Emerson, 1962), the level of leaders’ dependence will be influenced by their motivational investment to realize their goals and secure essential resources through subordinates. Leaders’ motivational investment can be shaped by individual differences in the power holder (Keng-Highberger et al., 2024) or contextual factors (i.e., status threat, G. Zhang et al., 2020). Leaders with a future-oriented personality tend to focus on the achievement of long-term goals and are more willing to perform behaviors with important distant consequences. Involving subordinates in task fulfillment and goal achievement can create advantages for the group or organization, ultimately helping leaders advance toward a better future, such as attaining higher status (Wee et al., 2017). As a result, these leaders are more motivated to value the input and contributions of their employees, leading to a stronger dependence on their subordinates.

Furthermore, highly future-oriented supervisors are likely to experience higher well-being (e.g., happiness) because they maintain a clearer and more optimistic view of their future. This, in turn, reduces worry about an uncertain future and lowers feelings of anxiety (Zheng & Ahmed, 2024). And then individuals with high well-being will develop a stronger sense of achievement from overcoming challenges, become more aware of their limitations, gain a deeper appreciation for the value of hard work, and are more willing to seek help. As a consequence, they are inclined to exhibit more humble actions (Tong et al., 2018). On the contrary, less future-oriented supervisors are more likely to dwell on past failures and disappointments (Kooij et al., 2017). They may focus on their subordinates’ shortcomings rather than their strengths, which can hinder collaboration. Such leaders may isolate themselves and exclude others from task execution. Finally, less future-oriented supervisors are less dependent on their subordinates’ contributions to achieve desired outcomes. Therefore, we propose:

**Hypothesis** **1.** *Supervisor future orientation is positively related to outcome dependence.*

### 2.3. Supervisor Outcome Dependence and Humble Leadership

The tenet of power dependence theory suggests that as leaders become more dependent on followers for contributions, expertise, and loyalty, their power becomes more vulnerable. In response, they take deliberate steps to consolidate and protect their authority (Emerson, 1962). Drawing from this theory, we argue that leaders’ outcome dependence on subordinates fosters humble leadership. Firstly, since leaders must take actions to meet group needs and accomplish work goals (Hackman & Walton, 1986), it is crucial for them to monitor their interactions with followers. They tend to develop a strong commitment to group performance and encourage subordinates to fully utilize their expertise and perform effectively. Consequently, supervisors need to exhibit cooperative behaviors and adopt a humble leadership style, as evidence suggests that high-status individuals who act cooperatively and humbly can motivate low-status individuals to reciprocate with cooperative behaviors (G. Zhang et al., 2020).

Moreover, when supervisors heavily rely on the work outcomes of their subordinates, they recognize that their own outcomes are closely tied to their subordinates’ performance (Xu & Li, 2024). In other words, their success depends on the efforts and contributions of their subordinates, and without these, their own effectiveness would be greatly diminished. Therefore, to stimulate the potential of their subordinates and achieve performance goals, supervisors are more likely to treat them with equality and respect, actively listen to their opinions, and acknowledge their achievements (Owens et al., 2013), thereby showing more humble leadership behaviors. Hence, we propose:

**Hypothesis** **2.** *Supervisor outcome dependence is positively related to humble leadership.*

### 2.4. Mediating Role of Supervisor Outcome Dependence

Based on the above arguments, we draw from power dependence theory and propose that a leader’s future orientation, which enhances their outcome dependence on subordinates, subsequently fosters humble leadership. Power dependence theory posits that, while leaders hold power over their followers, they are also dependent on their followers, as they require followers’ resources and input in the pursuit of their outcomes such as power consolidation and future goal achievement (Emerson, 1962). Therefore, future-oriented leaders prioritize the achievement of long-term goals and often face the need for additional resources as well as the challenge of tackling complex tasks (Kooij et al., 2017). Consequently, leaders with a strong future orientation tend to be more dependent on their subordinates for resources and input.

As their dependence on followers increases, their power becomes more vulnerable. To protect their authority and ensure the achievement of future goals, leaders tend to foster harmonious relationships with their team members (Lichtenthaler & Fischbach, 2019). They may adopt a more humble approach, and seeking cooperation through consultation, communication, and other collaborative means (Owens et al., 2013). Thus, supervisor outcome dependence may serve as a mediator between supervisor future orientation and humble leadership. Indirect evidence from W.-H. Zhang et al. (2014) suggests that leaders with a high future orientation may broaden their perspectives, attend to followers’ ideas and needs, and recognize the value of each follower. Based on these insights, we propose:

**Hypothesis** **3.** *Supervisor outcome dependence mediates the relationship between supervisor future orientation and humble leadership.*

### 2.5. Moderating Role of Supervisor Prevention Focus

As previously mentioned, future-oriented leaders tend to prioritize gains over losses when working toward a desired future, leading them to rely more on their subordinates and exhibit more humble behaviors. However, this process may not be fixed; rather, it may be contingent on other factors (Wang et al., 2024). We argue that a supervisor’s prevention focus will moderate the mediated relationship between supervisor future orientation and humble leadership. We focus on the moderating role of supervisors’ prevention focus rather than their promotion focus, as a promotion focus is closely associated with the pursuit of an ideal future and shares some conceptual overlap with future orientation (Pennington & Roese, 2003). Individuals with a prevention focus are primarily concerned with avoiding losses and securing their safety needs (Higgins, 1998). They tend to place greater emphasis on their duties and obligations, feel more tension and be less relaxed. Moreover, they are more likely to focus on proximate than distant tasks (Pennington & Roese, 2003).

From a regulatory focus perspective, the magnitude of outcome dependence experienced by future-oriented leaders is moderated by their prevention focus level. Specifically, when supervisors have a high prevention focus, they tend to be more concerned with avoiding negative outcomes and possible losses, and are more sensitive to potential risks and adverse situations (Higgins et al., 2003). In this case, supervisors with a high future orientation are in a greater need to progress smoothly toward their goals and avoid risks that may lead to failure (Stam et al., 2010). This leads supervisors to closely monitor distant tasks (Pennington & Roese, 2003), relying more on the participation and contributions of subordinates. Their cooperation helps minimize mistakes, reduce failures, and ensure goal achievement (Hackman & Walton, 1986).

In contrast, when supervisors have a low prevention focus, they tend to be more accepting of risks and less fearful of failure (Shi & Huang, 2022). Therefore, highly future-oriented supervisors with a low prevention focus are more relaxed in the pursuit of their long-term goals and are willing to explore all possibilities, they may not prioritize preventing high risks or the failure to achieve goals as much (Shi & Huang, 2022). Under such circumstances, they are less likely to perceive subordinates’ input as an effective and essential means of achieving goals and avoiding failure. As a result, they tend to rely less on their subordinates. Based on these insights, we propose:

**Hypothesis** **4.** *Supervisor prevention focus strengthens the relationship between supervisor future orientation and outcome dependence.*

Next, we argue that a leader’s prevention focus moderates the relationship between supervisor outcome dependence and humble leadership. According to power dependence theory (Emerson, 1962), the power relationship between leaders and subordinates is reciprocal. Consequently, in order to receive help from subordinates, who have a significant impact on their consequences, leaders need to be open to subordinates’ opinions, show respect and become humble.

Based on regulatory focus theory, such relationships may change as a leader’s prevention focus changes. When a supervisor’s prevention focus is strong, they are more focused on avoiding negative outcomes (Higgins et al., 2003). Supervisors who rely on their subordinates for success are particularly concerned that poor relationships with them may negatively impact results, thereby threatening their outcomes and positions within the organization (Xu & Li, 2024). To mitigate this risk, supervisors may adopt a more humble approach in their interactions, demonstrating affinity, respect and recognition (Owens et al., 2013; Zhong et al., 2023). This fosters subordinates’ active cooperation, reduces potential conflicts, and helps safeguard the stability of the supervisors’ outcomes and authority.

Conversely, supervisors with a low prevention focus are less sensitive to potential risks (Hamstra et al., 2011) and less attentive to negative cues in their environment (Fatimah et al., 2024). Even when supervisors are highly dependent on their subordinates for work-related outcomes, they may not be overly concerned that external risks will hinder their abilities to achieve desired results. They may perceive themselves as capable of managing uncertainties on their own or adapting to changing circumstances without needing to rely heavily on their subordinates. As a result, they are less inclined to adopt humble leadership behaviors, such as actively seeking subordinates’ input or securing their cooperation to address challenges (Chen et al., 2024). Based on the above reasoning, we propose:

**Hypothesis** **5.** *Supervisor prevention focus strengthens the relationship between supervisor outcome dependence and humble leadership.*

By combining the above hypotheses, we propose a moderated mediation model in which supervisor prevention focus influences the strength of the indirect relationship between supervisor future orientation and humble leadership via outcome dependence. Specifically, integrating power dependence theory and regulatory focus theory, when supervisors have a high prevention focus, they tend to be more concerned with avoiding negative outcomes and risks (Fatimah et al., 2024). In this context, highly future-oriented supervisors recognize that achieving long-term goals on their own may involve many challenges and uncertainties. Therefore, they are more likely to rely on the efforts of their subordinates to help achieve these goals. To motivate subordinates to fully realize their potential and engage actively in their work, supervisors need to adopt a humble attitude, such as listening to and recognizing their contributions. This fosters a sense of respect and value among subordinates (Zheng & Ahmed, 2024), increasing their motivation to invest effort and, ultimately, contributing to the realization of the supervisor’s future consequences.

On the contrary, when supervisors have a low prevention focus, they are less concerned about adverse situations. In this context, highly future-oriented supervisors are less concerned about potential risks and obstacles they may face when pursuing their outcomes. Moreover, they do not focus on avoiding failure, where involving subordinates’ contributions could be an important strategy. They tend to maintain a relatively positive and confident attitude (Pennington & Roese, 2003) when interacting with subordinates, and do not feel the need to display humility to gain their support or recognition. In summary, the following hypothesis is proposed:

**Hypothesis** **6.** *Supervisor prevention focus strengthens the mediating effect of supervisor outcome dependence on the relationship between supervisor future orientation and humble leadership.*

## 3. Method

### 3.1. Sample and Procedure

We conducted a quantitative, multi-wave field study. In this study, employees and their leaders from a large import and export company located in Southeast China were invited to participate in a questionnaire survey. Using a census sampling approach, we invited all available leader-subordinate dyads within the company to participate. We chose this industry because leaders and subordinates interacted frequently within this business. At the beginning of the investigation, the purpose of the study was explained, the voluntary nature of participation was emphasized, and it was assured that their personal information would be kept strictly confidential and that the data would be used solely for academic analysis. To make sure that questionnaires from both parties were matched, we assigned a unique number to each team based on a pre-acquired list, and then handed out separate packages on site. Data collection occurred over three waves, spanning approximately two months. Leaders were first asked to rate their future orientation, prevention focus and their demographic information. About three weeks later, they were invited to assess their outcome dependence on each subordinate. And three weeks later, these supervisors’ subordinates were asked to evaluate their perceptions of humble leadership and their own demographic information.

With the assistance of the company’s human resources manager, we sent 93 supervisor questionnaires, initially got 93 responses and ultimately kept 91 questionnaires, with two excluded due to a job transfer and extensive missing data, respectively. For the employee questionnaires, 215 were sent out, which was the total number of subordinates for these leaders in the company, and finally obtained 211 responses. After eliminating invalid questionnaires with incomplete responses, the final sample included 90 supervisor and 204 employee responses. The effective response rate of the questionnaires was 96.77% for leaders and 94.88% for employees. Among the supervisors, 47.80% were male, and their average age was 37.60 years old. In the sample of employees, 27.90% were male, the average age was 27.30 years old, and they had worked with their leaders for 3.68 years on average.

### 3.2. Measures

All of the scales used in the present study were originally developed in English. They were translated into Chinese following the standard translation and back-translation procedure (Brislin, 1980). Seven-point Likert scales were adopted for all main variables.

Humble leadership (α = 0.93). Employees evaluated their supervisors’ humble leadership according to the scale (1 = “strongly disagree”; 7 = “strongly agree”) developed by Owens and Hekman (2016), which contains eight items such as “This leader shows a willingness to learn from others.”

Future orientation (α = 0.76). Supervisor future orientation was assessed using Strathman et al.’s (1994) five-item scale (1 = “do not remember”; 7 = “often”). A sample item is “I think about future events and try to influence them with my daily behavior.”

Outcome dependence. Based on Walter et al.’s (2015) one-item scale (1 = “not dependent”; 7 = “fully dependent”), we asked leaders, “How dependent are you on this employee to achieve your work goals?” to measure their outcome dependence on subordinates. We adopted this concise, face-valid measure to minimize leaders’ cognitive load and enhance response quality for a conceptually straightforward construct, aligning with common practice in leadership research when measuring clear, unidimensional dependencies (Walter et al., 2015).

Prevention focus (α = 0.90). Supervisor prevention focus was rated using the six-item scale (1 = “strongly disagree”; 7 = “strongly agree”) developed by Wallace et al. (2009). This scale includes items like “I focus on following rules and regulations at work.”

Control variables. Referring to existing studies (Hoffman et al., 2011), we controlled for employees’ and leaders’ gender and age, as well as employees’ tenure with their leaders.

## 4. Results

### 4.1. Common Method Variance (CMV) Considerations

We employed a multi-wave, multi-source research design to proactively mitigate concerns regarding common method variance. Procedurally, we implemented several recommended safeguards. First, we collected data from different sources: supervisors provided ratings for their own future orientation and prevention focus, while their respective subordinates rated humble leadership. This physically separates the measurement of predictor and outcome variables. Second, we introduced a temporal separation of approximately three weeks between the measurement of the mediator (supervisor outcome dependence) and the dependent variable (subordinate-rated humble leadership), reducing the likelihood of transient common rater effects. Third, we guaranteed respondent anonymity to minimize social desirability bias. These design features collectively serve to substantially reduce the risk of common method bias in our study.

### 4.2. Measurement Model Results

Since the present study has just two subordinate-level variables (i.e., supervisor outcome dependence and humble leadership) and the former is measured by one item, the confirmatory factor analyses were conducted for the supervisor-level variables. Apart from the hypothetical two-factor model containing supervisor future orientation and prevention focus, a comparison model was created, specifically the one-factor model, which combines future orientation and prevention focus. The results showed that the two-factor model had a better fit to the data (χ^2^ = 48.79, df = 43, χ^2^/df = 1.13, RMSEA = 0.04, CFI = 0.99, TLI = 0.98) than the other model (χ^2^ = 137.18, df = 44, χ^2^/df = 3.12, RMSEA = 0.15, CFI = 0.79, TLI = 0.74), demonstrating that the two leader variables exhibited good discriminant validity.

### 4.3. Descriptive Statistics and Correlations

Table 1 presents the means, standard deviations, and correlations of our variables, in which supervisor outcome dependence was significantly and positively related to humble leadership (r = 0.21, *p* < 0.01), providing initial support for Hypothesis 2.

### 4.4. Hypotheses Testing

We next tested the proposed hypotheses and the results were displayed in Table 2. In line with Hypothesis 1, supervisor future orientation was significantly and positively correlated with outcome dependence (γ = 0.34, *p* < 0.01, Model 1). In Model 3, supervisor outcome dependence was significantly and positively related to humble leadership (γ = 0.16, *p* < 0.01); thus, Hypothesis 2 was also supported. To test the mediating role of supervisor outcome dependence, we utilized the Bootstrap method with 5000 iterations to produce a 95% level confidence interval and got supportive results (indirect effect = 0.06, 95% CI = [0.017, 0.125]) for Hypothesis 3.

**Figure 2:**
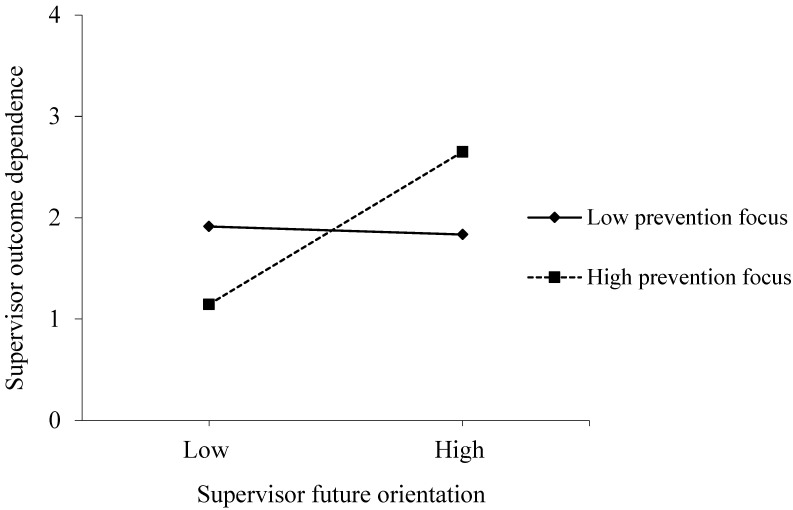
Moderating effect of supervisor prevention focus in the relationship between supervisor future orientation and outcome dependence.

Consistent with Hypothesis 4, Model 2 revealed that supervisor future orientation and prevention focus jointly and significantly influenced supervisor outcome dependence (γ = 0.40, *p* < 0.05). Furthermore, as shown in Figure 2, we depicted a simple slope plot to clarify the moderating effect. At a high level of supervisor prevention focus, supervisor future orientation still significantly promoted outcome dependence (t = 3.325, *p* < 0.01). When supervisor prevention focus was low, the relationship between the two variables was negative but not significant (t = −0.173, ns).

**Figure 3:**
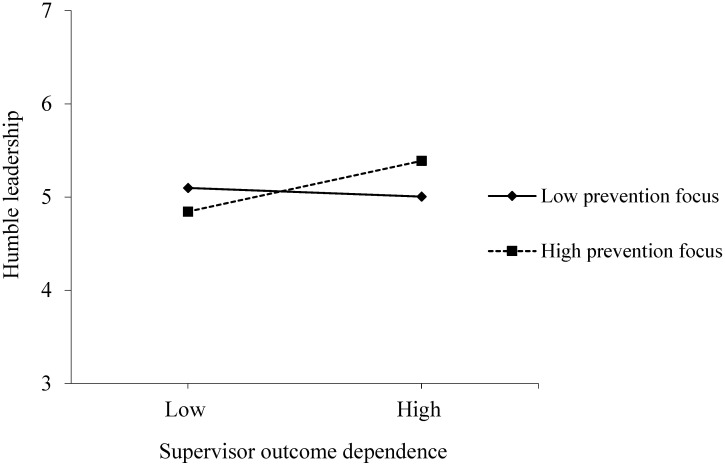
Moderating effect of supervisor prevention focus in the relationship between supervisor outcome dependence and humble leadership.

In addition, the interaction term involving supervisor outcome dependence and prevention focus significantly predicted humble leadership (γ = 0.16, *p* < 0.05, Model 4), and therefore, Hypothesis 5 was supported. As shown in Figure 3, we drew the corresponding simple slope plot to present this moderating effect. When supervisor prevention focus was high, supervisor outcome dependence significantly enhanced humble leadership (t = 2.414, *p* < 0.05). At a low level of supervisor prevention focus, the relationship between the two variables became non-significantly negative (t = −0.696, ns).

Further, using a conditional process analysis suggested by Hayes (2013), we tested for the moderated indirect effect of supervisor prevention focus. Results indicated that, under the condition of high supervisor prevention focus, the indirect influence of supervisor future orientation on humble leadership through outcome dependence remained significantly positive (indirect effect = 0.15, 95% CI = [0.039, 0.288]), but this effect became non-significant when supervisor prevention focus was low (indirect effect = 0.01, 95% CI = [−0.023, 0.069]). Moreover, the difference between the two conditions was significant (indirect effect = 0.14, 95% CI = [0.018, 0.278]). Taken together, Hypothesis 6 was supported.

## 5. Discussion

This study proposes a theoretical model that explains how future-oriented leaders rely on the work outcomes of their subordinates, leading to more humble behaviors and how leader prevention focus moderates such indirect relationship. Through data collected from supervisors and their immediate subordinates in a Chinese company, our study found that supervisors’ future orientation strengthened their outcome dependence, thereby resulting in a higher level of humble leadership. Moreover, supervisor prevention focus could reinforce the mediating effect of outcome dependence in the relationship between supervisor future orientation and humble leadership. Our findings, rooted in power dependence theory, suggest that humble leadership can emerge not only as a dispositional virtue but also as a strategic response to dependence and the need to manage relational risks to secure future outcomes.

### 5.1. Interpretation of Key Findings

Our core finding—that supervisor outcome dependence mediates the relationship between future orientation and humble leadership—advances the literature in two key ways. First, it unpacks the process underlying the observed link between leader future orientation and follower-focused behaviors (W.-H. Zhang et al., 2014) by identifying leaders’ perceived dependence on subordinates as a critical motivational mechanism. Second, it substantiates the rational choice view of humility (J. Yang et al., 2019), demonstrating that humility can be instrumental for leaders who depend on their subordinates to achieve important future outcomes.

Furthermore, the moderating role of supervisor prevention focus refines this instrumental account. The strengthened relationships when prevention focus is high suggest that the strategic display of humility is not merely a function of dependence, but is amplified by a leader’s concurrent motivation to avoid losses and secure those future outcomes. This integration of regulatory focus theory with our power-dependence framework reveals that humble leadership is most likely to emerge when leaders are driven by a combination of long-term aspirational goals and short-term risk-avoidance concerns.

### 5.2. Theoretical Contributions

Our findings make several contributions to the existing literature. First, we expand the nomological network of humble leadership by demonstrating that a leader’s future orientation positively predicts humble leadership behaviors through strengthening his or her outcome dependence on followers (Wang et al., 2018; J. Yang et al., 2019). Previous research has predominantly focused on the consequences of leader humility (Chandler et al., 2023; Jia et al., 2025; Zhong et al., 2023). A recent review suggests that research on individual factors as antecedents of humble leadership is still in its early stages and requires further exploration (Kelemen et al., 2023). Thus, the present research contributes by identifying supervisor future orientation and outcome dependence on subordinates as distal and proximal antecedents of humble leadership, respectively, offering new insights into how a leader’s personality trait in terms of future orientation shapes his or her humble leadership style. More importantly, it reframes leader humility, in part, as a pragmatically motivated behavior elicited by power-dynamics and risk sensitivity, complementing the predominant view of humility as a stable character strength.

Second, this study makes a significant contribution to the emerging literature on future orientation by highlighting that a leader’s future orientation can enhance his or her outcome dependence, thereby resulting in a higher frequency of humble leadership behaviors. Previous studies on future orientation have mainly adopted a lower-level perspective and focused on the positive effects of employees’ future orientation on their own actions such as increased task performance (Kooij et al., 2017; Lee et al., 2017). However, the effects of a leader’s future orientation on leadership have received limited attention. In addition, while it has been reported that a leader with a high future orientation is likely to show care for followers’ ideas and needs (W.-H. Zhang et al., 2014), the underlying mechanism involved is unclear. As such, the present study’s exploration of the relationship between supervisor future orientation and humble leadership, as well as its examination of the mediating role of supervisor outcome dependence, not only enriches the understanding of the effects of a leader’s future orientation on leadership but also reveals its underlying mechanism.

Furthermore, this study introduces a novel theoretical perspective to explain why future-oriented leaders exhibit humble leadership actions. Previous studies have primarily employed theories such as the implicit theory of self and rational choice theory to understand why leaders display humble behaviors (Wang et al., 2018; J. Yang et al., 2019). That is, leaders may exhibit humility when they have a growth mindset or when their subordinates are competent. Indeed, according to power dependence theory (Emerson, 1962), leaders are dependent on their subordinates, and the consequences of their subordinates are in part related to what the leaders can achieve (Johnson & Johnson, 1989). Therefore, leaders are more likely to display humility toward their subordinates because these subordinates have a significant impact on the achievement of their own goals. However, no empirical research has yet explored this linkage. As the first study to apply power dependence theory to explain the mediating role of supervisor outcome dependence between supervisor future orientation and humble leadership, our findings contribute to a more comprehensive understanding of the process through which humble leaders are formed.

Finally, by revealing the moderating role of supervisor prevention focus, this study enriches the knowledge of the boundary conditions affecting the formation of humble leadership. Previous research has shown that authority centralization (Peng et al., 2020) and leader relational identity (Wang et al., 2024) moderate the development of a leader’s humility. Integrating regulatory focus theory with power dependence theory, we find that supervisors’ prevention focus strengthens the influence of their future orientation on humble leadership through outcome dependence. This study unveils how it integrates with power dependence theory and the temporal personality literature to influence the emergence of leadership.

### 5.3. Practical Implications

This study offers several practical implications for organizations. First, our findings show that a leader’s future orientation positively predicts humble leadership. Thus, organizations should recruit or promote management candidates who have a high level of future orientation, so that they are more likely to show humility (Zheng & Ahmed, 2024). Managers can test candidate leaders’ tendency toward humility by asking situational questions. Additionally, HR managers can offer leadership training and development programs to teach leaders to fully understand themselves, show respect to subordinates and appreciate others’ strengths.

Second, our study found that a leader’s outcome dependence could enhance his or her humility level. Thus, managers should empower supervisors and encourage them to actively involve employees in team performance. Moreover, organizations should consider linking leader performance with subordinates’ work outcomes. When setting leaders’ performance appraisal objectives, organizations could place greater emphasis on team collaboration and encourage leaders to strengthen their reliance on subordinates for achieving collective goals. They should also focus on improving employees’ skills and capabilities, thereby increasing their contributions to the team (G. Zhang et al., 2020).

Last but not least, it is important to note that future-oriented supervisors exhibit diverse levels of humble leadership behaviors due to their prevention focus level. This study showed that a leader’s prevention focus amplified the positive impact of the leader’s future orientation on humble leadership through outcome dependence. Thus, organizations could help managers develop a higher level of prevention focus by clearly conveying work goals and expectations. Managers should actively assess and monitor the level of leaders’ prevention focus to ensure the conditions needed for fostering humble leadership are in place.

### 5.4. Limitations and Future Research Directions

It is important to note that this study still has some limitations that require further exploration. First, although we invited leaders and their followers to measure all variables at two time points, we were still unable to determine the causal relationships between supervisor future orientation and humble leadership through outcome dependence. We call for future research to employ longitudinal or experimental designs, which can further strengthen the persuasiveness of the causal relationships among our focal variables.

Second, the sample for this study was drawn exclusively from a single company in Southeast China, thus limiting the generalizability of our findings. Previous studies have shown that, compared to Western leaders, Chinese leaders are more likely to display humility, influenced by Confucian values emphasizing interpersonal harmony (Jia et al., 2025). Additionally, Chinese culture tends to emphasize a long-term orientation, which may further shape leadership behaviors and employee responses differently from those in short-term-oriented cultures. Therefore, our findings may vary across cultures and should be applied cautiously in other settings. We encourage future cross-cultural research to test these relationships in different countries (Wen et al., 2025).

Finally, like many humble leadership studies (Wang et al., 2018; Zheng & Ahmed, 2024), this study invited employees to evaluate their leaders’ humility, and utilized Owens et al.’s (2013) scale. Chandler et al. (2023) suggested that to improve the accuracy of leader humility measurement, additional sources such as peers, the leader and the leader’s superior should be included, and alternative humility scales could be used. Therefore, future research could incorporate diverse sources and measures to assess leader humility, offering a more comprehensive and nuanced understanding of the factors shaping humble leadership.

## 6. Conclusions

Based on power dependence theory and regulatory focus theory, this study explains why a supervisor’s future orientation leads to humble leadership. Findings from a multi-wave, multi-source field study are consistent with the model proposing that a supervisor’s future orientation is associated with his or her outcome dependence on subordinates, which, in turn, is linked to more humble leadership behaviors. Moreover, supervisor prevention focus not only strengthens the positive effect of future orientation on outcome dependence, but also reinforces the positive relationship between supervisor outcome dependence and humble leadership. Additionally, the mediating role of supervisor outcome dependence in the relationship between supervisor future orientation and humble leadership is stronger when a supervisor’s prevention focus is high. We hope that these findings contribute to a deeper academic and practical understanding of how and under what conditions a supervisor’s individual differences predict humble leadership.

## Figures and Tables

**Table 1:** Descriptive statistics and intercorrelations.

Variable	M	SD	1	2	3	4
Subordinate-level variables						
1. Subordinate gender	1.72	0.45				
2. Subordinate age	2.26	0.98	−0.13			
3. Tenure with the supervisor	3.34	1.21	−0.04	0.37 **		
4. Supervisor outcome dependence	4.50	1.37	0.04	−0.02	0.14	
5. Humble leadership	5.41	0.96	0.06	−0.10	0.04	0.21 **
Supervisor-level variables						
1. Supervisor gender	1.52	0.50				
2. Supervisor age	4.32	1.35	−0.15			
3. Supervisor future orientation	5.56	0.81	−0.02	−0.00		
4. Supervisor prevention focus	6.25	0.65	0.03	0.03	0.38 **	

Notes. N(Subordinate) = 204; N(Supervisor) = 90. ** *p* < 0.01.

**Table 2:** Results of hierarchical regression analysis.

Predictors	Supervisor Outcome Dependence		Humble Leadership	
	Model 1	Model 2	Model 3	Model 4
Subordinate gender	0.26	0.24	0.02	0.05
Subordinate age	−0.24 *	−0.20	−0.10	−0.12
Tenure with the supervisor	0.20 *	0.19 *	0.04	0.06
Supervisor gender	0.06	0.11	0.14	0.14
Supervisor age	0.43 ***	0.43 ***	−0.04	0.03
Supervisor future orientation	0.34 **	0.36 **		
Supervisor outcome dependence			0.16 **	0.11 *
Supervisor prevention focus		0.01		0.03
Future orientation × prevention focus		0.40 *		
Outcome dependence × prevention focus				0.16 *

Note. N(Subordinate) = 204; N(Supervisor) = 90. * *p* < 0.05; ** *p* < 0.01; *** *p* < 0.001.

## Data Availability

The data presented in this study are available on request from the corresponding author. The data are not publicly available as they contain information that could compromise the privacy of research participants and the confidentiality agreement with the partner organization.
